# Marginal and internal adaptation of different flowable composite restorations in class V cavities after thermomechanical cyclic loading: in vitro study

**DOI:** 10.1186/s12903-025-07324-0

**Published:** 2025-12-09

**Authors:** Sahar A. Saleh, Maha M. Ebaya, Ashraf I. Ali

**Affiliations:** 1 Conservative Dentistry Department, Faculty of Dentistry, Mansoura University, Mansoura, Egypt; 2 Faculty of dentistry, New-Mansoura University, New-Mansoura, Egypt

**Keywords:** Class v cavity, Flowable composite, Internal adaptation, Marginal adaptation, Thermomechanical

## Abstract

**Objective:**

This in vitro study aimed to evaluate and compare the marginal and internal adaptation of five different flowable composite restorations in Class V cavities after thermomechanical cyclic loading.

**Materials and methods:**

One hundred freshly extracted human premolars were selected. Standardized Class V cavities were prepared on the facial surface, with gingival margin located 1 mm coronal to cementoenamel junction. Teeth were randomly assigned into five groups (*n* = 20) according to the restorative material: Group I: Short fiber-reinforced flowable( NanovaPro Flow); Group II: Self-adhesive flowable(Vertise Flow); Group III: ORMOCER-based bulk-fill flowable(Admira fusion X-base); Group IV: Resin-based bulk-fill flowable(Venus Bulk Flow); Group V: Injectable flowable(Gaenial Universal Injectable). Each group was subdivided (*n* = 10/ subgroup) for evaluation either at baseline (Non-Thermomechanical cyclic loading, N-TMC) or after thermomechanical cyclic loading (TMC).The TMC protocol involved 5000 thermal cycles (5°±1℃ to 55°±1℃) and 100,000 cycles of mechanical loading (100 N, 4 Hz). Marginal and internal adaptation were evaluated using a scanning electron microscope (SEM) at 200x magnification, with gaps > 1 μm considered defective. Data were statistically analyzed using the Monte Carlo test to compare the studied groups, and the McNemar test was used to compare N-TMC and TMC results, with a statistically significant level set at (*P* ≤ 0.05).

**Results:**

At baseline (N-TMC), all groups showed 100% perfect marginal adaptation (< 1 μm gap), with no significant differences between them (*p* = 1.0). After TMC, a significant deterioration in marginal adaptation occurred in all groups (*p* ≤ 0.007 for all within-group comparisons), with no significant differences between the groups (*p* = 0.08). For internal adaptation, there was a statistically significant difference in N-TMC results when comparing groups with *p* < 0.001, and no statistically significant difference between the groups after TMC *p* = 1.0. Finally, there was a statistically significant difference when comparing N-TMC with TMC in all groups with *p* < 0.001 except group V, where there was no statistically significant difference with *P* = 1.0.

**Conclusions:**

Within the limitations of this in vitro study, while all materials demonstrated good initial marginal and internal sealing, thermomechanical cycling significantly impaired the adaptation of both marginal and internal interfaces. The short fiber-reinforced flowable composite exhibited the most favorable performance, showing the least degradation after aging. This finding suggests that this material offers a promising solution for increasing the durability of Class V restorations.

## Introduction

 Restoring class V cavities is a common yet challenging clinical procedure, with reported failure rates for composite restorations ranging from 5% to30% over 5 years, primarily due to its proximity to the gingiva and challenges with moisture control. This can result in inadequate bonding to the cavity walls and gap formation between the restoration material and the tooth. Additionally, the shrinkage forces of resin-based composites can cause interfacial microleakage, potentially leading to marginal discoloration, secondary caries, or loss of retention [1]. 

The variations in filler content of resin composites influence whether the material is sculptable or flowable. Flowable composites have a lower filler load and viscosity, which reportedly enhances wettability and adhesion to cavity surfaces and walls. Moreover, these composites feature a lower elastic modulus, leading to increased flexibility and better resistance to flexural stress at the tooth cervix [1, 2]. 

However, in the past years, bulk-fill resin-based composites (BF-RBCs) have been introduced to the dental market to address challenges associated with the incremental technique for posterior teeth. Initially, the term bulk-fill referred to resin composites that permitted 4–5 mm increments, suitable for full-body and base bulk-fill techniques [2]. 

Additionally, dental restoration adaptation and thermomechanical cycling are crucial for restorative materials’ long-term success, impacting durability and function. However, thermomechanical cycling, subjecting materials to temperature changes and mechanical stress, can affect their performance. These cycles cause thermal expansion and contraction, potentially creating gaps between the restoration and tooth structure and leading to wear and fatigue in the material [3]. This degradation compromises the seal, causes discomfort, or leads to the failure of restoration [4]. Clinical evaluations of restorations present challenges due to ethical considerations, cost and time requirements [5]. In vitro studies that simulate oral conditions have been conducted to estimate restoration longevity.

The marginal seal of composite restorations represents an increasing concern. Over the years, in vitro evaluations of the performance of resin adhesives revealed that microleakage and gap formation, mainly at the dentin-composite interface, did not improve at the same rate as did bond strength values Independent of the bonding capacity of an adhesive system, it seems that adhesive restorations are far from assuring a perfect marginal seal, with degradation in time occurring regardless of the product used [6, 7]. Marginal and internal integrity results from several parameters related to the forces created by curing contraction, as bond strength alone could not be correlated to the adaptation [8]. Finally, Furthermore, upon reviewing the literature, there are few studies concerning the marginal and internal adaptation of bulkfill-flowable composite restorative systems after thermomechanical cycling have been published, and more data are still required. Therefore, this study was designed to answer the following research question: How do different flowable composite restorative materials behave in terms of marginal and internal adaptation in Class V cavities before and after being subjected to thermomechanical aging? Based on this, the null hypothesis tested was that there would be no significant difference in the marginal and internal adaptation among different flowable composite restorative materials in Class V cavities before and after thermomechanical cycling.

### Materials

This study was conducted after obtaining ethical approval from the faculty of dentistry’s ethical committee under No. (A03012023CD). The sample size calculation was based on a previous study on marginal adaptation [9]. Using the G power program version 3.1.9.7 to calculate sample size based on an effect size of 1.7, a 2-tailed test, α error = 0.05, and power = 85.0%, the total estimated sample size is 7 in each subgroup, which was increased to 10 for a more robust result.

### Specimen selection and Preparation

One hundred freshly extracted human premolars from the oral surgery clinic, Faculty of Dentistry, Mansoura University, were selected for this study. They were extracted for orthodontic reasons. The teeth were carefully inspected using light to ensure they were free from caries, restorations, cracks, or other defects. Soft tissue remnants were removed using a hand scaler (Zeffiro; Lascod, Florence, Italy). Teeth were stored in 1% chloramine-T for 48 h and subsequently in distilled water that was changed weekly until use.

**Fig. 1:**
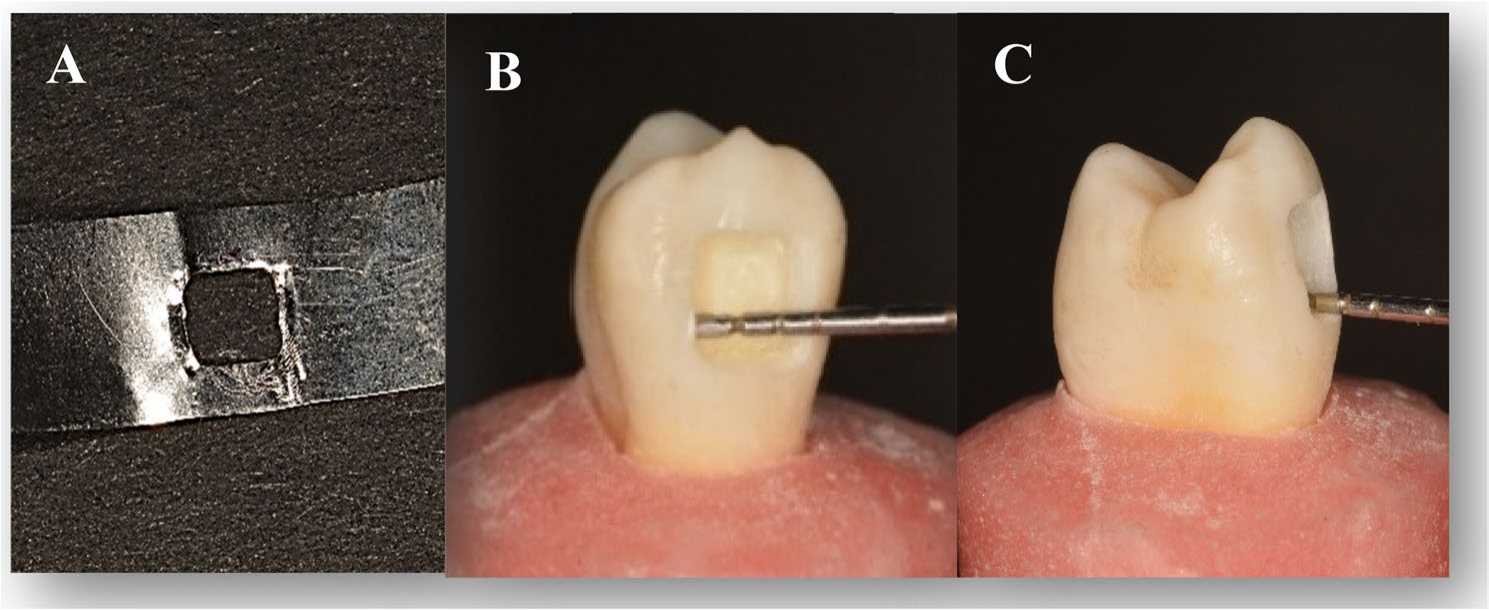
Showing **A** standardized using a stainless steel matrix band; **B** cavity measurement mesiodistally **C** depth of prepared cavity

Following teeth selection, the roots of all teeth were coated with a thin layer of polyvinyl siloxane impression material to simulate the periodontal ligament. Each tooth was then mounted vertically up to the CEJ in a self-curing acrylic resin block within a cylindrical mold, ensuring the facial surface with the cavity was exposed and parallel to the base for standardized loading. Class V cavity with measurements 3 mm mesiodistal (width), 3 mm occluso-gingival (height), and 2 mm axial (depth) was prepared on the facial surface of each tooth with gingival margin located 1 mm coronal to cementoenamel junction(CEJ) to standardize the substrate to enamel for all margins and eliminate dentin/cementum as a variable in this initial comparison of materials [10]. The surface angles were kept at 90 degrees without bevel designs [11]. The outline of the cavities was standardized using a stainless steel matrix band. Figures 1 preparation was performed with a straight-fissure diamond bur no. (SF-41 MANI Ltd., Utsunomiya, Japan) with a water-cooled high-speed handpiece (30,000–50,000 rpm, NSK, Shinagawa City, Japan). Bur was replaced after every five preparations [11].

### Specimen Grouping

**Fig. 2:**
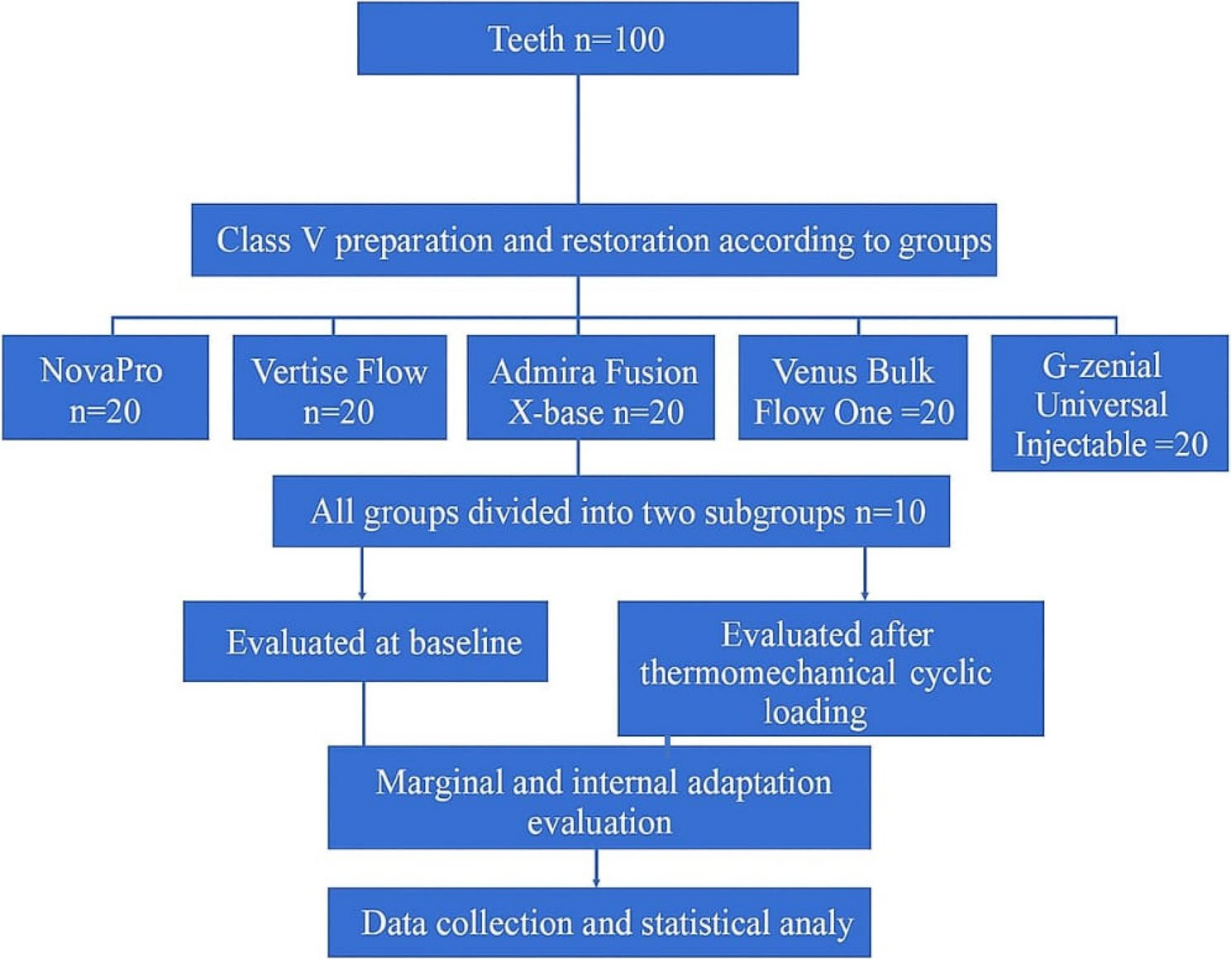
Diagram that illustrates the experimental design and grouping

Specimens were randomly divided into five groups based on restorative materials (*n* = 20) using an online randomizing program (www.randomizer.com). Group I was restored with NovaPro Flow (Nanova, Columbia, MO, USA), Group II with Vertise Flow (Kerr, USA), Group III with Admira Fusion X-base (Voco GmbH, Cuxhaven, Germany), Group IV with Venus Bulk Flow One (Kulzer GmbH, Germany), and Group V with G-ænial Universal Injectable (GC Co., Tokyo, Japan). Groups were further divided into two subgroups (*n* = 10) based on thermomechanical cycling: Subgroup I was examined at non-thermomechanical cycling (N-TMC), and Subgroup II was examined after thermomechanical cycling (TMC) with 5,000 cycles at 5°±1℃ and 55°±1℃, with dwell times of 60 s and transfer times of 15 s. Using the thermocycling machine (SD Mechatronic thermocycler, Germany) and for mechanical stimulation, a total of 100,000 cycles of an occlusal load at 100 N and 4 Hz were applied (SD Mechatronic CS-4, Germany), which represents 6 months in the oral environment [12]. Figure. 2.

### Restoration Procedures

All restoration procedures were performed strictly according to the manufacturers’ instructions. The adhesive systems used for each group were as follows:

- Groups I, III, IV, V: A universal adhesive system (Scotchbond, 3 M ESPE) was used. Enamel was etched with 35% phosphoric acid (Scotchbond etchant, 3 M) for 15 s, rinsed for 15 s, and gently air-dried. A single coat of the adhesive was applied with a microbrush for 15 s, rubbed in for 20 s gently air dried for 5 s, and then light-cured with Light emitting diode (LED) unit for 10 s (Woodpecker Co. Ltd. China) with a light intensity of 1200 mW/cm². The irradiance was checked at baseline before ap⁸plying the adhesive system and restorative materials using a radiometer (Bluephase Meter, Ivoclar Vivadent AG, Schaan, Liechtenstein) in all testing groups.
- Group II (Self-adhesive): No separate adhesive was used. A thin layer of the self-adhesive flowable composite (Kerr, USA) was dispensed using a small brush then the material was scrubbed for 15 s, and light cured for 20 s then the remaining cavity was filled and light cured according to manufacturer instructions.

Subsequently, the cavities were restored with the assigned flowable composites and light-cured according to their respective manufacturers’ recommended protocols.

### Evaluation marginal adaptation

All teeth are mounted on aluminum stubs and then coated with gold using a sputter coater (Sputter Coating Evaporator, SPI Supply, USA). They were examined under a Scanning electronic microscope (JSM-6510LV, JEOL Ltd., Tokyo, Japan) at magnification x25-200 [13, 14]. All SEM examinations and measurements were conducted by a single operator experienced in quantitative margin analysis and unaware of the restorative materials. Detectable gaps were checked and calculated at ×200, and images were analyzed using image analysis software (SEM Control User Interface Ver 3.10, JEOL Ltd.). The interface between each material and the substrate was also quantitatively analysed. The marginal adaptation was scored 0 if the interface between the restoration and tooth was continuous and exhibited less than 1 μm gap and scored 1 if the interface had gaps more than 1 μm wide [15]. 

### Internal adaptation evaluation

Teeth were sectioned longitudinally with a slow-speed diamond saw (Isomet 4000-Buehler, Lake Bluff, IL, USA) with water coolant in a buccolingual direction. Each specimen received 1 cut to produce 2 slices per specimen. Then, it was mounted on aluminum stubs and coated with gold using a sputter coater (Sputter Coating Evaporator, SPI Supply, USA). The inner side of each slice’s material/dentin interface was evaluated for internal adaptation using SEM using the same technique and parameters mentioned in the marginal adaptation part. The inner side of the restorative material/dentin interface was evaluated for internal adaptation using SEM using the same technique and parameters mentioned in marginal adaptation [13]. 

### Statistical analysis

Data analysis was performed using SPSS software, version 26 (Chicago: SPSS Inc.). Qualitative data were described using numbers and percentages. The significance of the obtained results was judged at a P.≤ 0.05. The Monte Carlo test was used to compare between studied groups, and the MC Nemar test was used to compare N-TMC and TMC results.

## Results

### Marginal adaptation

Examining marginal integrity with SEM in N-TMC or delayed subgroups for comparison among different groups, we observe gaps with all materials of varying sizes in the following order: short fiber-reinforced flowable with very small gaps, followed by resin-based bulk-fill flowable, ORMOCER-based BFF, self-adhering flowable and lastly, conventional flowable composite. All groups demonstrated 100% perfect marginal adaptation at baseline, with no statistically significant differences (*p* = 1.0). After TMC, a significant deterioration occurred in all groups. There was no statistically significant difference between the groups after aging (*p* = 0.08) and gaps > 1 μm.

Beyond the quantitative scoring, a qualitative assessment of the SEM micrographs was performed to identify the most prevalent locations of gap formation. This analysis revealed a consistent pattern. For Marginal Adaptation, interfacial gaps were most frequently observed along the gingival and lateral (mesial and distal) walls. The occlusal margin generally exhibited better integrity with fewer and smaller gaps.

**Fig. 3:**
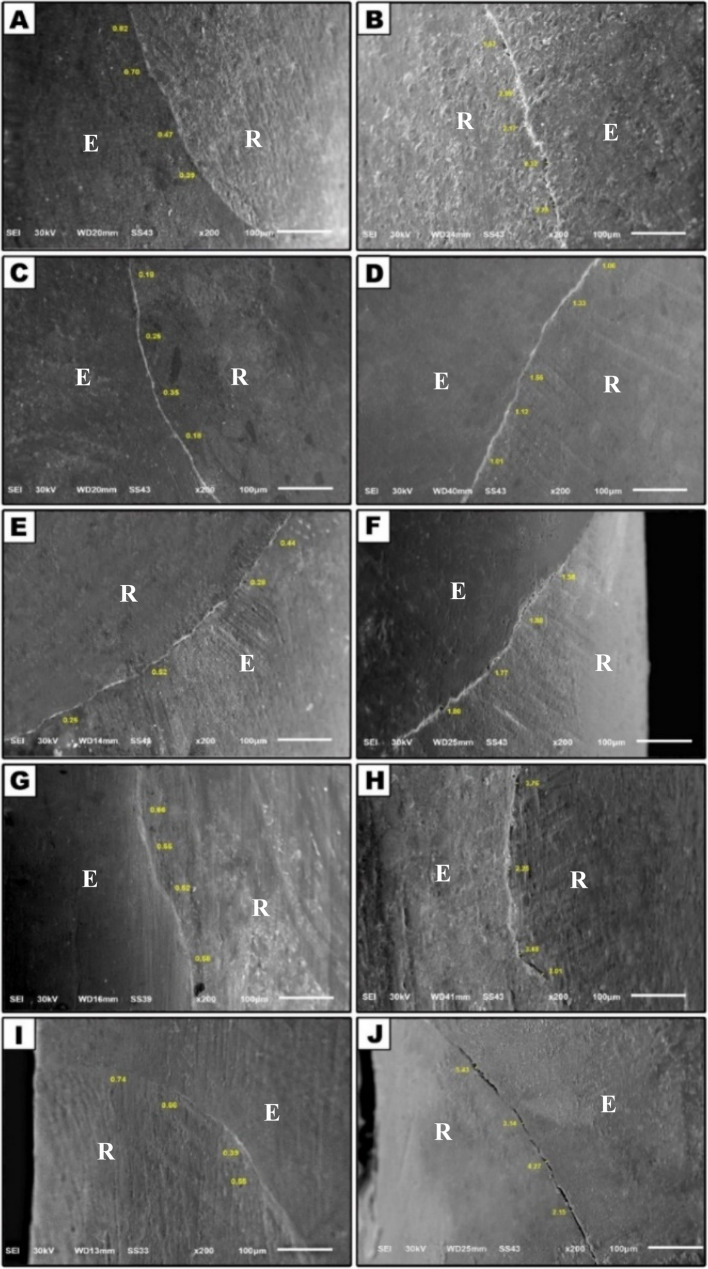
SEM photomicrographs showing marginal adaptation at the 200X magnification the left picture N-TMC examination; right after TMC **A B** the ORMOCER-based BFF **C D** fiber-reinforced flowable **E F **bulk-fill flowable **G** **H** self-adhering flowable **I J** conventional flowable

In other ways, these gaps are statistically represented when comparing the materials in the same groups, both at N-TMC and in TMC state. A clear and significant difference exists across all groups, indicating an increase of more than 1 μm gaps. All groups show 100%, except for the short fiber-reinforced flowable group, which has 80% with a gap greater than 1 μm and 20% with an intact margin of less than 1 μm, and this supported the SEM finding. As shown in Table 1 and Figure 3.

**Table 1:** Comparison of the marginal adaptation between studied groups between N-TMC versus TMC

Marginal Adaptation	ORMOCER-based BFF *N* = 10 (%)	Short fiber reinforced F *N* = 10(%)	BFF *N* = 10(%)	Self-adhering flowable *N* = 10(%)	Injectable flowable composite *N* = 10(%)	Test of significance (Monte Carlo test) and Effect size
N-TMC < 1 μm > 1 μm	10(100) 0	10(100) 0	10(100) 0	10(100) 0	10(100) 0	*P* = 1.0 η² = 0.984
TMC < 1 μm > 1 μm	0 10(100)	2(20) 8(80)	0 10(100)	0 10(100)	0 10(100)	*P* = 0.08 η² =0.975 Mc = 8.33
MC Nemar test	*P* < 0.001*	*P* = 0007*	*P* < 0.001*	*P* < 0.001*	*P* < 0.001*	η² =0.994
Effect size(Mean Difference [95%CI])	−1.92(−2.02, −1.82)	−1.09(−1.44, −0.74)	−1.43 (−1.57, −1.29)	−2.84(−3.10, −2.58)	−2.96(−3.1, −2.82)	

*Statistically significant (*P* < 0.05), *η²* Partial Eta Squared; *CI* Confidence Interval

### Internal adaptation

Examining internal adaptation integrity with SEM at N-TMC or TMC subgroups for comparison among different groups, we observe gaps with all materials of varying sizes in the following order: short fiber-reinforced flowable with very small gaps, followed by bulk-fill flowable, ORMOCER-based BFF, self-adhering flowable and lastly, injectable flowable composite. These observations were reported as 100% in all groups except injectable flowable composite 10% in the N-TMC examination, with statistically significant differences (*P* < 0.001*) with a gap of less than 1 μm. Furthermore, no statistically significant difference exists between groups in the TMC state, with *P* = 1.0 and more than 1 μm gaps.

A quantitative analysis of the internal adaptation interfaces revealed that the majority of gaps were predominantly located at the junction between the restoration and the axial (pulpal) wall, particularly the cavity line angles where the axial wall meets the gingival and lateral walls.

**Fig. 4:**
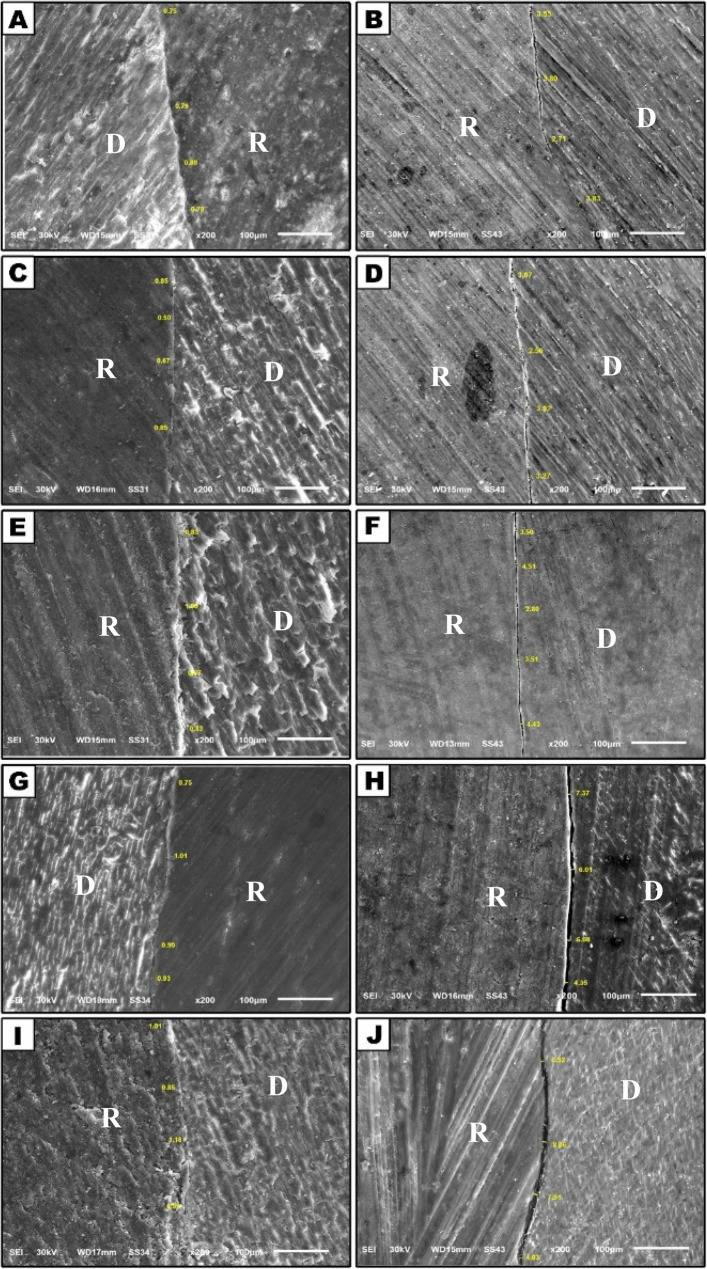
SEM photomicrographs showing internal adaptation at the 200X magnification the left side at N-TMC examination; right side after TMC **A** **B** the ORMOCER-based BFF **C** **D** short fiber-reinforced F **E F** BFF **G H** self-adhering flowable **I** **J** injectable flowable

In other words, when comparing the material in the same group N-TMC and the TMC examination, there is a clear significant difference across the first four groups with *P* < 0.001*, indicating a gap increase of more than 1 μm. At the same time, with injectable flowable composite, there is no statistically significant difference with *P* = 1.0, and these support the SEM finding. As shown in Table 2 and Figure. 4.

**Table 2:** Comparison of internal adaptation between studied groups and between N- TMC versus TMC

Internal Adaptation	ORMOCER-based BFF *N* = 10 (%)	Short fiber reinforced F *N* = 10(%)	BFF *N* = 10(%)	Self-adhering flowable *N* = 10(%)	Injectable flowable composite *N* = 10(%)	Test of significance (Monte Carlo test) and Effect size
N-TMC < 1 μm > 1 μm	10(100) 0	10(100) 0	10(100) 0	10(100) 0	1(10) 9(90)	Mc = 43.90 *P* < 0.001* η² =0.526
TMC < 1 μm > 1 μm	0 10(100)	0 10(100)	0 10(100)	0 10(100)	0 10(100)	*P* = 1.0 η² =0.395
MC Nemar test	*P* < 0.001*	*P* < 0.001*	*P* < 0.001*	*P* < 0.001*	*P* = 1.0	η² =0.939
Effect Size(Mean Difference[95%CI])	−1.94[−2.61,−1.27]	−1.76[−3.38,−0.14]	−1034[−2.85,−0.17]	−1.4[−2.07, −0.73]	−0.9[−2.85, 1.05]	

*Statistically significant (*P* < 0.05); *η² * Partial Eta Squared, *CI* Confidence Interval

## Discussion

Class V cavities are characterized by their location on the gingival third of the teeth. This poses unique challenges for restorative dentistry, including moisture control, adhesion to enamel, and the potential for high mechanical and thermal stresses due to their proximity to the gingival tissues [16]. Restoration success in a class V cavity depends mainly on the material’s ability to adapt marginally and internally to the cavity walls, ensuring a durable seal that prevents microleakage and secondary caries [17]. Restorative materials must also withstand occlusal and lateral forces while maintaining biocompatibility and esthetics in this visible dentition area. Advances in restorative materials, particularly bulk-fill flowable composites, have shown promise in overcoming these challenges by improving ease of application, adaptation properties, and mechanical performance [18]. 

Selecting an appropriate resin composite and adhesive system in the class V cavity is crucial for restoration success. The main challenge in restoring this type of cavities is ensuring proper marginal adaptation [19]. Polymerization shrinkage (PS) during resin curing induces internal stress at the tooth-restoration interface, weakening the bond in flexible, porous dentin. This can cause marginal gaps or microleakage, allowing fluids or bacteria to enter, leading to inflammation, secondary caries, or pulp issues. Shrinkage-induced stress can weaken the adhesive and tooth bond, especially when the dentin is too wet [17]. 

Bulk-fill flowable materials in class V cavities improve clinical outcomes by reducing postoperative sensitivity and extending restoration longevity. BFF materials address challenges in class V restorations with lower PS, minimizing marginal gaps and reducing the risk of fractures or adhesive failure. Their flowable properties allow close conformation to cavity wall margins [20]. This enhances internal adaptation and reduces the need for multiple layers, minimizing voids that could weaken the restoration. BFF materials streamline the process, lowering error rates and increasing efficiency. Advanced BFF matches the mechanical properties of traditional composites, making them suitable for stress-bearing areas of class V cavities [21]. 

This study used a box-shaped preparation to standardize experimental groups. The consistent cavity configuration minimized variations in preparation techniques, ensuring restorations were evaluated under similar conditions. This approach facilitated reliable comparisons by controlling for differences in cavity geometry that could affect restorative procedure outcomes [22]. In Class V cavities, achieving ideal adaptation is particularly challenging due to the higher C-factor inherent in these designs.

Scanning Electron Microscopy (SEM) is a vital tool for assessing the marginal adaptation of dental materials. It generates high-resolution images of the tooth-restoration interface, allowing detailed examination of gaps and defects by evaluating the fit, surface morphology, and bonding quality. This helps to identify issues that may affect longevity [23]. Direct sample analysis using SEM is preferred over replicas, as it avoids replication artifacts and ensures an accurate interface representation [24, 25]. 

This method provides relevant data by examining the materials without alterations from the replica-making process. Overall, SEM accurately evaluates the restoration’s performance, including potential gaps and bacterial or saliva penetration risk [26]. Gaps of less than 1 μm are generally associated with better marginal and internal adaptation [15]. This reduces bacterial penetration and longer-lasting restorations. However, even gaps at this small scale should be minimized to prevent long-term risks. More significant gaps (greater than 1 μm) significantly increase the potential for bacterial penetration, leading to secondary caries, marginal breakdown, and restoration failure. Therefore, achieving precise marginal and internal adaptation during restorative procedures is essential for the durability and success of dental restorations [27]. 

Gloria Kang GJ et al. [27] Investigated the effect of bacterial penetration into gaps. It was shown that smaller gap samples had less bacterial penetration. When more significant gaps exist, bacteria easily penetrate to the full depth of the gap, regardless of loading conditions, increasing the incidence of secondary caries formation.

This study executed 5,000 thermal cycles to evaluate the direct correlation with the increase in marginal gaps at the dental restoration interface [28, 29]. After that, specimens underwent 100,000 cycles of 100 N occlusal load at 4 Hz [29]. This equates to six months of clinical functionality, assuming that these cycles happen 10 to 25 times a day, as many studies suggest [28, 30]. The mechanical simulator provides insights into a material’s performance during prolonged use and how dental restorations react to complex forces in the oral cavity by simulating stress distribution, fatigue, wear, and crack propagation. The simulator considers the physiological traits of human chewing and the direction of jaw movements [12]. Thermal cycling affects the bonding agent’s or adhesive’s properties, while mechanical cycling, such as forces from chewing, may stress the bond interface [31]. 

This study showed that all groups tested at baseline achieved 100% marginal adaptation integrity without significant differences between groups. Short fiber-reinforced flowable composites (SFRC-F) showed the best results among groups, which may be due to the content of hydroxyapatite fibers, which are particularly beneficial in enhancing bioactivity and mineralization. Hydroxyapatite, a primary component of tooth enamel, can chemically interact with the enamel surface, providing stronger interfacial adhesion due to its similar composition. The presence of hydroxyapatite fibers helps improve the chemical bonding between the composite and enamel, providing excellent initial bond strength.

In the BFF resin-based composite group, the matrix comprises multifunctional methacrylate monomers, UDMA, EBADMA, and Bis-EMA. These monomers can achieve a good bond with enamel through the chemical interaction of the monomers with enamel’s hydroxyapatite. Also, its flowability allows the composite to fill micro-irregularities in the enamel and cavity walls, ensuring micromechanical retention. This helps the material bond more effectively with the tooth surface by getting into tiny pores or grooves and creating a strong mechanical lock.

ORMOCER-based BFF inorganic component is chemically incorporated into the organic polymer, meaning that the material is inherently a hybrid and the two phases (organic and inorganic) are more interconnected at a molecular level, leading to enhanced properties such as reduced shrinkage and more substantial marginal adaptation. The organic resin portion allows the material to bond to the enamel through functional monomers with acid-functional groups such as Bis-EMA, aliphatic dimethacrylate, and UDMA, which can interact with enamel. These monomers can bond chemically to the enamel’s mineral content, creating a chemical adhesive bond between the ORMOCER composite and the tooth structure. At the same time, the silicon oxide nanofillers and glass ceramics filler give the material additional durability, strength, and wear resistance, which is crucial for achieving a tight marginal seal. These materials are designed to form substantial, well-sealed restoration with excellent marginal adaptation, reducing the risk of bacterial infiltration and ensuring the long-term success of the restoration.

Furthermore, Self-adhesive flowable composites are designed with chemicals that allow them to bond to enamel surfaces chemically. These materials contain functional monomers (like carboxyl and phosphoric acid groups) capable of interacting with the mineral components in enamel, even without the need for etching. These monomers help form a bond with the tooth structure by chemically interacting with hydroxyapatite, the main component of enamel [32]. 

Injectable flowable material had good marginal adaptation integrity, possibly due to its high flow, meaning low viscosity. This allows it to adapt to enamel surfaces and fill intricate cavities easily. It also penetrates micro-irregularities and creates close contact with the enamel while maintaining a smooth surface.

Also, Agarwal et al. [33] Finds that all tested materials showed acceptable marginal adaptation in enamel before TMC. Unfortunately, this level of adaptation declined after TMC. In addition, Tonetto et al. [12] Demonstrated that TMC obstructed the marginal adaptation of enamel in every group compared to the initial conditions, and both agreed with our findings. Furthermore, this aligns with the results of Abdelwahed et al. [34] Who stated that no group, regardless of the restorative materials utilized, achieved 100% continuous margins.

According to the SEM assessment after TMC group, the SFRC-F group showed less discontinuous margin to the enamel, followed by the BFF group, then the ORMOCER-based BFF group, then self-adhesive flowable, and finally injectable flowable.

However, there was no statistically significant difference among groups in the delayed examination. Only the SFRC-F maintained marginal integrity, with 20% of specimens exhibiting this quality. This may be due to the incorporation of short fibers in flowable composites significantly reducing PS. The presence of short fibers helps the resin retain its structural integrity during the curing process, minimizing the shrinkage process [35]. SFRC-F typically have a higher content of fillers. The filler system in this material, usually made from silica and/or barium glass, plays an equally important role in the material’s cyclic aging resistance. These fillers contribute to wear resistance, providing abrasion resistance and helping the material maintain its integrity during the masticatory cycle. This is especially important for preventing marginal deterioration after the material undergoes repeated mechanical stress. The dimensional stability of inorganic fillers helps maintain the composite’s shape and structure during thermal cycling (heating and cooling), preventing excessive expansion or contraction. This helps avoid forming marginal gaps that could lead to microleakage or secondary caries [36]. 

A study by Roggendorf et al. [37] Stated that gap-free margins were primarily found under thermomechanical stress conditions. And ElAziz et al. [38] Who evaluated flowable short fiber-reinforced flowable composite restorations compared to conventional packable composites and found no significant differences between the groups, noting an increase in gaps in marginal integrity after 6 and 12 months.

The BFF resin-based composite group showed open enamel margins after cyclic aging, likely due to the fluoride-releasing Ytterbium Fluoride (YbF3). Fluoride promotes hydrolytic degradation of the resin matrix, increasing water sorption in the composite over time. This can lead to resin softening and ultimately result in bond degradation at the resin-enamel interface, causing margin failure. Additionally, chemical interactions between fluoride and the composite may further destabilize the bond under long-term moisture exposure and cyclic aging. Moreover, barium glass fillers enhance the mechanical properties of composites but may affect bonding enamel. Fillers can stress the resin-filler interface if poorly bonded to the resin matrix. Cyclic loading, like chewing, may debond fillers, leading to failure at the composite-enamel interface and weakening overall strength adhesion.

Moreover, the ORMOCER-based BFF group had large filler particles like glass ceramic fillers with a particle size of 1 μm) which can reduce the material’s flexibility, making it more prone to stress concentration. While nanofillers improve surface smoothness and mechanical properties, their presence with larger fillers can reduce adaptability to the enamel surface during polymerization and cyclic loading. High filler loading typically enhances mechanical properties like strength and wear resistance. Still, exceeding a threshold (e.g., 70–80%) can increase the material’s viscosity, which may negatively affect the composite’s wetting ability on the enamel surface. If the resin fails to wet and adapt to the enamel surface adequately, it may not form a strong bond. Marginal gaps can form due to incomplete interface filling.

In addition, the self-adhesive flowable incorporated with pre-polymerized fillers are typically added to improve the composite material’s strength and wear resistance. However, their addition can affect the polymerization process and the adaptation to the enamel. If the polymerization of the pre-polymerized fillers is not perfectly coordinated with the surrounding matrix material, it could result in incomplete polymerization or weak spots within the resin matrix. These weak spots can lead to microcracks or debonding at the interface, contributing to the development of marginal gaps. Furthermore, the mechanical loading from chewing forces may exacerbate any pre-existing micro gaps and cause marginal failure. The combination of methacrylate monomers and fillers, particularly YbF3, can also enhance hydrolytic degradation at the enamel-resin interface after extended exposure to moisture, as observed in the oral cavity. Water absorption within the resin matrix produces softening and swelling, which may cause the bond to disintegrate. Cyclic aging, which simulates the thermal and mechanical stresses during mastication, can expedite this process, ultimately forming marginal gaps.

Injectable flowable composites with barium and strontium fillers have different thermal expansion coefficients (CTE) than enamel. Thermomechanical cycling, which simulates the changes in temperature (from hot to cold) and the mechanical forces from chewing, can cause both the composite and the enamel to expand and contract at different rates. A CTE mismatch increases stress at the resin-enamel interface during thermal cycling, leading to marginal gaps or debonding. This issue worsens if the composite is overly filled with inorganic materials like barium glass strontium.

De Albuquerque Jassé et al. [39] supported our results when evaluated the marginal adaptation before and after TMC of the BFF and conventional composite resin. A significant improvement in marginal adaptation was observed when BFF was used instead of traditional composite resin before and after TMC. The results of this study confirm that the BFF-RBCs tested have characteristics comparable to or superior to those of conventional resin regarding marginal adaptation. However, only long-term clinical trials can confirm the clinical success of the material.

On the other hand, internal adaptation was preserved in all groups during the baseline examination except for the conventional flowable composite. We noted a gap in 90% of specimens in that group at baseline examination, likely due to the inconsistent hybrid layer formation. Low filler content, moisture sensitivity, and limited adhesive penetration into dentin affect traditional flowable composites. Dentin surfaces are treated less intensively, and improper conditioning, such as inadequate smear layer disruption, can prevent optimal bonding. The smear layer can block adhesive penetration and negatively impact bonding. Moreover, low-viscosity flowable composites have higher shrinkage rates due to reduced filler, risking separation from tooth structure and diminishing adhesion and marginal adaptation.

The SFRC-F group’s adhesive’s inability to properly penetrate dentin due to a lack of demineralization results in weak bonding to dentin. Several factors can contribute to this, mostly its weak formulation, which is missing many strong adhesive compounds such as MDP and other methacrylates. Its weak acidity also results in poor adhesive penetration. In conjunction with the selective etching technique, this could result in more significant gaps in dentin after thermocycling aging and force loading on the bonding interface.

ORMOCER-based BFF group showed internal gaps mainly due to Diethyl amino benzaldehyde (DEAB), used as an accelerator in adhesive polymerization, which can contribute to weakening the dentin bond over time, especially after cyclic aging. One of the key factors is oxidative degradation, as DEAB can undergo oxidation in the presence of oxygen, particularly when exposed to moisture. This oxidation can interfere with the polymerization process, leading to incomplete curing and reduced bond strength. Additionally, DEAB is water-soluble and may leach into the oral environment, causing hydrolytic degradation of the adhesive bond when exposed to saliva and moisture. This leaching can further compromise the bond’s durability. Moreover, while DEAB aids in speeding up polymerization, it may not effectively contribute to the crosslinking of the polymer network, resulting in a weaker matrix that is more susceptible to thermal cycling and mechanical stress, ultimately leading to a weakened dentin bond.

The BFF resin-based composite group contains hydroxyethyl methacrylate (HEMA), a hydrophilic monomer used in its adhesive system to enhance wetting and penetration into dentin. While HEMA improves initial bond strength, it can pose problems over time, especially under cyclic aging. Due to its hydrophilic nature, HEMA can cause water absorption into the adhesive layer. Over time, this moisture can lead to hydrolytic degradation of the resin-dentin interface and result in marginal leakage. HEMA’s softening effect on the resin can make the bond more vulnerable to mechanical stresses, such as thermal cycling and masticatory forces, weakening the bond over time. Alongside methacryloxyethyltrimellitic acid anhydride (4-META) monomer, which is also used in this adhesive, reacts with dentin to form chemical bonds. However, its sensitivity to moisture can also result in hydrolytic degradation under cyclic aging conditions.

Self-adhering flowable showed internal gaps, possibly due to glycerol phosphate dimethacrylate (GPDM), a newer monomer in self-adhering composites. It enhances chemical bonding to dentin by reacting with hydroxyapatite and forming a stable bond to the substrate. However, after cyclic aging, GPDM may contribute to bond failure due to phosphate groups, which are susceptible to hydrolytic degradation when exposed to water over time. Moisture infiltration at the interface can cause the phosphate ester bonds to break down, leading to bond failure at the resin-dentin interface.

Ultimately, internal adaptation revealed gaps exceeding 1 μm across all groups during the delayed evaluation. There were statistically significant differences in all groups except conventional flowable composite. Dentin is a porous tissue that contains water and organic materials. The resin matrix formed with Bis-EMA, UDMA, and dimethacrylate monomers is relatively hydrophobic; this moisture could impair the resin’s capacity to create a strong bond with dentin, which resulted in discontinuous internal margins before the aging process. Moreover, cyclic aging typically replicates temperature fluctuations (thermal cycling) and mechanical stress (chewing forces), which can lead to the expansion and contraction of the resin. Consequently, water absorption and demineralization can weaken the adhesive bond over time if the resin is hydrophobic and does not adhere effectively to dentin. Following cyclic aging, the bond may deteriorate further due to water infiltration at the interface.

A study by Roggendorf et al. [37] Agreed with this study and found a larger gap formation internally after TMC. Also Tonetto et al. [12] Demonstrated that TMC obstructed the adaptation of dentin in every group compared to the initial conditions. In contrast Karabekiroglu et al. [8] observed that TMC doesn’t have an adverse effect on the dentin bond strength of most adhesive systems in Class V cavities. Also, El Naga et al. [40], the dentin integrity and BFFs were compared with conventional RBCs, which were used in both bulk filling techniques. The study’s results revealed that the BFF restorations demonstrated a similar marginal gap formation to conventional universal RBCs under their research conditions.

In the same way as our result, Elhawary et al. [41] a comparison of BFF and conventional flowable composites showed that the BFF composite has lower microleakage scores than the traditional flowable composite at both occlusal and gingival margins. This may be due to the established multifunctional methacrylate monomers, such as UDMA (urethane dimethacrylate) and EBADMA (ethoxyethyl methacrylate), which provide strong bonding to the tooth structure and resistance to stress, both of which are crucial for the longevity of restorations.

In contrast to our study, Maj et al. [42] conducted a comparative clinical study on the Self-adhering flowable composite and the traditional flowable composite. After six months, they found visible superficial damage to the margins of the filling in almost all cases of the Self-adhering flowable, while most cases with the traditional flowable composite exhibited better marginal integrity simultaneously. These differences may be due to the methodology used. Also, Baltacioğlu et al. [43] Compared to composite groups with low viscosities, the conventional flowable exhibited the lowest leakage, with no statistically significant difference from the BFF group.

The current study’s findings indicate that thermomechanical cycling affects the marginal and internal adaptation of all materials with statistically significant differences among all groups. In addition to the quantitative results, a qualitative assessment of the SEM micrographs revealed a consistent pattern across most groups: the majority of marginal gaps were observed at the gingival, while the occlusal margin generally exhibited better integrity. For internal adaptation, gaps were most frequently located at the interface with the axial wall and the cavity line angles. This pattern can be attributed to the high cavity configuration factor (C-factor) of Class V preparations, which exacerbates polymerization shrinkage stress. The resulting tensile forces are concentrated at the most constrained areas of the restoration often overcoming the bond strength and leading to debonding. The thermomechanical cycling likely accelerated this failure by fatiguing the bonded interfaces at these vulnerable sites [3, 6, 44]. 

This contrasts with Casselli et al. [6] evaluated the effect of TMC, margin location, and the adhesive system on the marginal adaptation of Class V cavities restored with micro-hybrid resin composite restorations. They concluded that the TMC did not alter the gap measurements and did not affect marginal adaptation.

According to the results of this study the null hypothesis states that the type of restoration didn’t affect the marginal adaptation at baseline examination has been accepted. The type of restoration that did not affect the marginal adaptation during the delayed examination has been rejected. The type of restoration did not affect the internal adaptation during the baseline examination. On the other hand, the type of restoration did not impact the internal adaptation during the delayed examination, and this result has been rejected.

Several limitations inherent to this in vitro study must be acknowledged. First, while thermomechanical cycling provides a valuable simulation of oral conditions, it cannot fully replicate the complex biological environment, including salivary flow, variable pH, and enzymatic activity. Second, while the cavity design within enamel was essential for isolating the variable of material under optimal bonding conditions, it limits the direct clinical extrapolation of the results, as Class V restorations often involve the more challenging dentin/cementum substrate at the gingival margin. Furthermore, the assessment of marginal and internal adaptation provided an overall score for the entire interface. A future quantitative analysis measuring gap width and length at specific cavity walls could provide even more detailed insight into the localization of failure. Finally, the study evaluated a single time point after aging; longer-term degradation patterns remain unexplored. These factors should be considered when interpreting the results.

## Conclusions

Within the limitations of this in vitro study, while all materials demonstrated good initial marginal and internal sealing, thermomechanical cycling significantly impaired the adaptation of both marginal and internal interfaces. The short fiber-reinforced flowable composite exhibited the most favorable performance, showing the least degradation after aging. This finding suggests that this material offers a promising solution for increasing the durability of Class V restorations.

### Recommendations

Different cavity designs and sizes are needed to test the effectiveness of the tested materials and confirm the current study’s results. Further research is required, utilizing different thermomechanical cycling durations. More in vivo studies should be conducted to evaluate and compare the clinical performance of the tested materials. Using a 3D tool like micro-CT for measuring marginal and internal gaps can provide a superior assessment compared to 2D techniques. It evaluates not just the width and length of the gaps, but also their depth.

## Data Availability

The datasets used and/or analyzed during the current study are available from the corresponding author on reasonable request.

## Abbreviations

BFF: Bulk Fill flowable
BF-RBCs: Bulk-fill resin-based composites
CEJ: Cementoenamel junction
CM: Continuous margin
DC: Degree of conversion
DEAB: Diethyl amino benzaldehyde
EBADMA: ethoxyethyl methacrylate
Fig.: Figure
GPDM: Glycerol phosphate dimethacrylate
HEMA: Hydroxyethyl methacrylate
MDP: Methacryloyloxydecyl dihydrogen phosphate
MA: Marginal adaptation
SEM: Scanning electron microscope
TMC: Thermo-mechanical cycling
UDMA: Urethane dimethacrylate
µm: Micrometer
